# The Value of Primary Tumor Resection in Patients with Liver Metastases: A 10-Year Outcome

**DOI:** 10.1245/s10434-024-16386-3

**Published:** 2024-11-04

**Authors:** Lin-Lin Liu, Yu-Kun Lin, Zuo-Lin Xiang

**Affiliations:** Department of Radiation Oncology, Shanghai East Hospital, Tongji University School of Medicine, Shanghai, China

**Keywords:** SEER, Primary tumor resection, Liver metastases, Nomogram

## Abstract

**Objective:**

This study aimed to analyze the impact of primary tumor resection (PTR) on the prognosis of four common primary tumors with liver metastases, and to develop a prognostic model to visualize the PTR benefit rate of patients with liver metastases.

**Materials and Methods:**

Patients diagnosed with colorectal cancer liver metastases (CRLM), pancreatic cancer liver metastases (PLM), gastric cancer liver metastases (GLM), and breast cancer liver metastases (BLM) between 2004 and 2015 were retrospectively reviewed from the Surveillance, Epidemiology, and End Results (SEER) database and assigned to either the surgery or non-surgery groups. A 1:1 propensity score matching (PSM) was performed. Surgical patients who survived longer than the median cancer-specific survival (CSS) time for non-surgery patients constituted the benefit group. Logistic regression was conducted to explore the independent factors affecting surgical benefit, and a nomogram was established.

**Results:**

A total of 21,928 patients with liver metastases were included. After PSM for surgery and non-surgery patients, we found that PTR had a significant impact on the overall survival (OS) and CSS of CRLM, PLM, and BLM patients. In CRLM patients, age (*p* < 0.001), primary site (*p =* 0.006), grade (*p* = 0.009), N stage (*p* = 0.034), and histology (*p* = 0.006) affected the surgical benefit. In BLM patients, the independent factors were age (*p* = 0.002), race (*p* = 0.020), and radiotherapy (*p* = 0.043). And in PLM patients, chemotherapy was an independent factor associated with a survival benefit from PTR.

**Conclusion:**

PTR improved OS and CSS in patients with CRLM, PLM, and BLM. A predictive model was established to identify suitable candidates for PTR in CRLM patients.

**Supplementary Information:**

The online version contains supplementary material available at 10.1245/s10434-024-16386-3.

The liver is a common site of tumor metastases, with up to 50% of various cancer patients experiencing liver metastases during the occurrence and development of disease.^1^ This is partly due to the unique and diverse cellular and structural composition of liver, which confers a high affinity for tumor cells. Data from the Surveillance, Epidemiology, and End Results (SEER) database indicate that 5.1% of patients have synchronous liver metastasis when diagnosed with primary cancer. This proportion of patients varies according to the type of primary cancer, from 0.3% of prostate cancer patients to 35% of pancreatic cancer patients.^2^ A variety of malignant tumors, including primary tumors originating in the colorectum, gastrointestinal tract, pancreas, and breast, typically exhibit liver metastasis.

The management of liver metastases patients is complex, therefore implementing effective and tailored treatment methods is crucial. According to the current guidelines of the National Comprehensive Cancer Network (NCCN), systemic therapy is the mainstream treatment method for patients with metastatic malignant tumors, while primary tumor resection (PTR) is still at a low recommended level;^3^ however, studies have shown that PTR can affect the prognosis. According to current guidelines, surgical resection combined with chemotherapy is the recommended treatment for resectable colorectal cancer liver metastases (CRLM) patients, while chemotherapy is the recommended treatment for non-resectable patients.^4^ Analysis of data from patients from six European pancreatic centers showed that patients who underwent combined liver pancreatic resection had significant survival benefits compared with those who did not undergo primary pancreatic ductal adenocarcinoma resection for liver metastasis (14.5 months vs. 7.5 months; *p *= 0.001).^5^ For gastric cancer liver metastases (GLM) patients, when surgery is unavoidable, the guidelines consider palliative gastrectomy as an option.^6^ Over the years, some observational studies have shown that 35–60% of patients with stage IV breast cancer receive primary tumor treatment at the time of initial diagnosis, which can benefit survival. The European Society for Medical Oncology (ESMO) guidelines suggest that the role of PTR in stage IV breast cancer is not clear at present and that PTR should be considered in specific patients.^7^ In this situation, how to select suitable PTR patients has become a question worth considering.

Therefore, it is necessary to clarify whether PTR can benefit the survival of patients with liver metastases. Meanwhile, the extent and influencing factors of surgical benefit need to be highlighted. This study focuses on the four types of tumors that are prone to liver metastasis, using the SEER database. Patients with liver metastases were studied at a population-based level. Our study provides appropriate information to support the choice of surgical options and quantifies the benefit of PTR.

## Methods

### Data Extraction

The SEER database is a definitive cancer statistics database that records the information of people with malignant tumors in the United States, and can be publicly used for cancer-based epidemiological research and survival analysis. We submitted the data agreement form to the SEER administration, and the data were obtained using SEER*Stat version 8.3.5 (accessed on 10 February 2021). The requirement for ethical approval was waived by the Institutional Review Committee because the SEER database is freely available to global researchers.

### Data Arrangement

**Fig. 1:**
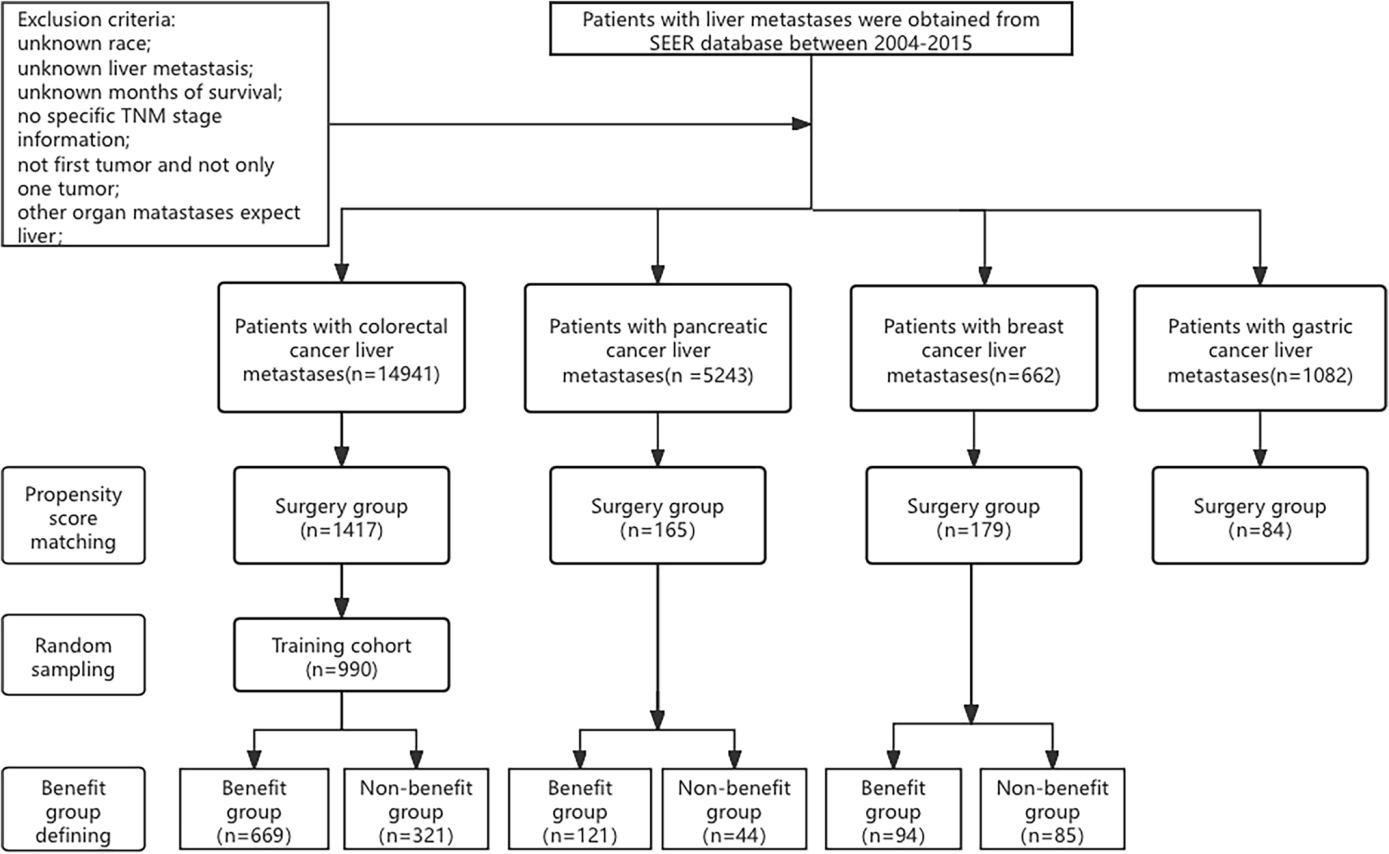
Study selection process. *SEER* Surveillance, Epidemiology, and End Results

Newly diagnosed cases of CRLM, pancreatic cancer liver metastases (PLM), GLM, and breast cancer liver metastases (BLM) were extracted from the SEER database from 2004 to 2015. Pathological diagnoses of colorectal adenocarcinoma, pancreatic ductal adenocarcinoma, gastric adenocarcinoma, signet ring cell carcinoma, breast infiltrating duct carcinoma, and lobular carcinoma were included. PTR referred to wide excision of the primary lesion but did not include local tumor destructive methods such as photodynamic therapy, cryosurgery, electrocautery, laser ablation, and excisional biopsy. Based on the recorded variable ‘RX Summ–Surg Prim Site (1998+)’, cases with PTR were identified. The exclusion criteria were (1) unknown liver metastasis; (2) not the first tumor and not the only tumor; (3) no specific TNM stage information; (4) unknown race; (5) unknown months of survival; and (6) combined with other organ metastases. According to the above principles, we selected the enrolled population (Fig. 1). For each record, we calculated demographics, clinical variables, and follow-up data.

Based on the cause of death, we divided patients into two categories: cancer death and non-cancer death. Overall survival (OS) refers to the time from a confirmed diagnosis of a malignant tumor to death caused by any reason; the last follow-up date was considered to be the end date if the patient had not died by the follow-up deadline. Cancer-specific survival (CSS) refers to the time from the diagnostic date to the date of death from a specific cancer. We defined the benefit group as surgical patients with a longer CSS than the median CSS of the non-surgery group, and therefore classified the surgery population into two groups—the benefit group and the non-benefit group, based on the above assumption.

### Statistical Analysis

Descriptive statistics were applied to elucidate the baseline characteristics of patients with liver metastases. According to whether PTR was performed, patients were categorized into two groups. Propensity score matching (PSM) is a practical method to reduce confusion bias. The age, histology, race, sex, grade, T stage, N stage, primary site, chemotherapy, and radiotherapy of patients in the surgery and non-surgery groups were matched through non-replacement 1:1 matching with calipers, as a 0.05 standard deviation of the logit of the propensity score. Before and after PSM, the Chi-square test was used to test the significance of each clinical feature difference between the surgery and non-surgery groups. The OS and CSS of patients in the surgery and non-surgery groups before and after PSM were calculated using the Kaplan‒Meier method and estimated using the log-rank test. In the surgery group, the Chi-square test was used to compare the benefit rates of each variable between the benefit and non-benefit groups to evaluate the risk factors that could benefit from PTR. Variables selected with statistical significance (*p * < 0.05) were included in logistic regression. The cases in the surgery group were randomly sampled, grouped at a ratio of 7:3, and named the training and validation sets, respectively. A nomogram was established using the training cohort based on binary logistic regression. Discriminant and calibration curves were constructed internally (training cohort) and externally (validation cohort) to predict the accuracy of the nomogram, and performance was measured using the Harrell consistency index (C-index). Statistical analysis was performed using SPSS 26.0 (IBM Corporation, Armonk, NY, USA) and R 4.2.1 (https://www.r-project.org/). All statistical tests were two-tailed, and *p *< 0.05 was considered statistically significant.

### Practical Applications of the Nomogram in the Clinical Setting

Based on the produced nomogram, we calculated the probability of benefit from PTR for those in the surgery group. Patients were classified according to the following two levels: if the total predicted probability was >0.5, the patient was classified as a candidate for surgical benefit, whereas participants with a total predicted probability ≤0.5 were classified as candidates who would not benefit from surgery.

### Patient Data from Our Center

Our study included 34 CRLM patients who underwent PTR at Shanghai East Hospital between May 2019 and March 2022. The inclusion criteria were (1) age older than 18 years; (2) diagnosed with CRLM by pathological biopsy; (3) magnetic resonance imaging (MRI) was used to confirm liver metastases; and (4) more than 2 years of follow-up. Demographic and clinical data were collected from each record. This study followed the Declaration of Helsinki and the ethical principles of Shanghai East Hospital.

## Results

### Patient Characteristics and Propensity Score Matching

From 2004 to 2015, the proportion of patients with CRLM, PLM, GLM, and BLM receiving PTR was 70.3%, 3.3%, 11.9%, and 45.6%, respectively. As shown in electronic supplementary material (ESM) Tables 1–4, there were disparities in certain baseline variables between the surgery and non-surgery groups (*p* < 0.05). To balance these factors and attain comparability between the two groups, liver metastases patients with or without PTR were separately matched through PSM. After PSM, the baseline features between the two groups were well-balanced (all *p* > 0.05) [ESM Tables 1–4].

### Survival Outcomes in Liver Metastases Patients with or without Surgery

**Fig. 2:**
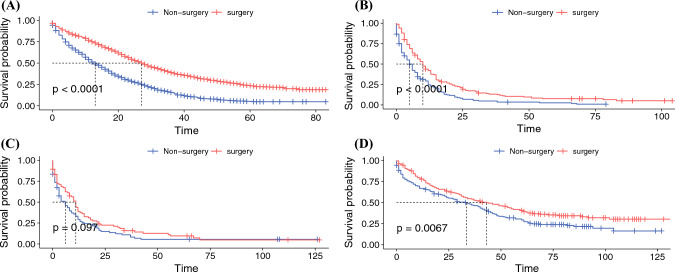
Cancer-specific survival plots of (**A**) CRLM, (**B**) PLM, (**C**) GLM, and (**D**) BLM patients, according to treatment after PSM. *CRLM* colorectal cancer liver metastases, *PLM* pancreatic cancer liver metastases, *GLM* gastric cancer liver metastases, *BLM* breast cancer liver metastases, *PSM* propensity score matching

**Fig. 3:**
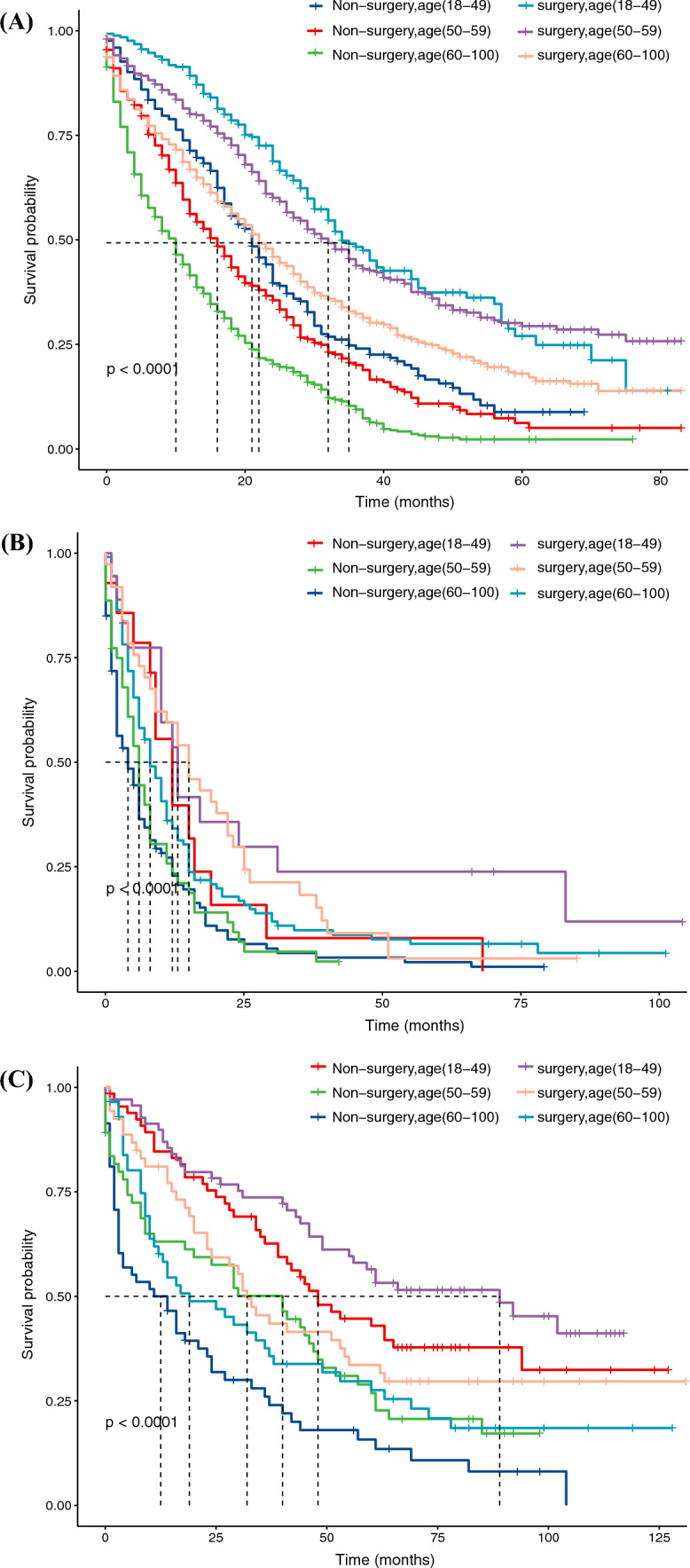
Cancer-specific survival plots of (**A**) CRLM, (**B**) PLM, and (**C**) BLM patients, according to age and treatment after PSM. *CRLM* colorectal cancer liver metastases, *PLM* pancreatic cancer liver metastases, *BLM* breast cancer liver metastases, *PSM* propensity score matching

After PSM, the 1- and 3-year OS rates of the surgery group versus the non-surgery group were as follows: CRLM: 75.0% vs. 51.3%, 38.9% vs. 15.4%; PLM: 40.6% vs. 22.0%, 11.8% vs. 4.3%; GLM: 37.4% vs. 28.8%, 13.3% vs. 10.7%; and BLM: 75.9% vs. 65.9%, 52.1% vs. 44.1%, respectively. The surgery group had a longer median OS and CCS compared with the non-surgery group, both before and after PSM (Table 1). As shown in Fig. 2, Kaplan–Meier analysis showed significant differences in CSS between the surgery and non-surgery groups among patients with CRLM, BLM, and PLM. Due to the consideration that the surgical benefits of young people are higher than those of the elderly, age-stratified analysis was conducted to evaluate the surgical benefits of different age groups. As shown in Fig. 3, the CSS of the surgery group was longer than that of the non-surgery group across all age categories. Therefore, it is suggested that PTR can be beneficial for individuals of various age groups with CRLM, BLM, and PLM.

**Table 1:** Median survival time of patients according to treatment before and after propensity score matching

		Median OS (months)		Median CSS (months)	
		Surgery (95%CI) vs. non-surgery (95%CI)	*P* value	Surgery (95%CI) vs. non-surgery (95%CI)	*P* value
CRLM	Before PSM	21 (20-21) vs. 10 (9-10)	<0.001	22 (21-22) vs. 11 (10-11)	<0.001
	After PSM	27 (25-28) vs. 13 (11-14)	<0.001	24 (23-25) vs. 12 (10-12)	<0.001
PLM	Before PSM	10(8-12) vs. 3 (3-3)	<0.001	10 (8-12) vs. 3 (3-4)	<0.001
	After PSM	10 (8-12) vs. 5 (4-6)	<0.001	10 (8-13) vs. 5 (4-7)	<0.001
GLM	Before PSM	8 (6-11) vs. 5 (4-6)	<0.001	9 (6-11) vs. 5 (5-6)	<0.001
	After PSM	10 (8-12) vs. 6 (3-9)	0.190	11 (8-13) vs. 6 (3-9)	0.097
BLM	Before PSM	48 (37-57) vs. 25 (18-29)	<0.001	53 (43-63) vs. 29 (23-36)	<0.001
	After PSM	40 (31-53) vs. 29 (22-40)	0.004	43 (32-59) vs. 34 (24-42)	0.007

*OS* overall survival, *CSS* cancer-specific survival, *CRLM* colorectal cancer liver metastases, *PSM* propensity score matching, *PLM* pancreatic cancer liver metastases, *GLM* gastric cancer liver metastases, *BLM* breast cancer liver metastases

### Factors Affecting the Surgical Benefits of Patients with Liver Metastases

In CRLM, the median CSS time (12 months) in the non-surgery population was selected as the time point, and the surgical population was then divided into two groups: the benefit group (CSS time ≥12 months) and the non-benefit group (CSS time <12 months). The median CSS time in the non-surgery population of PLM and BLM was 5 months and 34 months, respectively. To explore factors influencing the benefit of PTR, we conducted univariate analysis and binary logistic regression to identify the independent risk factors (Table 2). In CRLM, factors associated with the benefit of PTR included age (odds ratio [OR] 0.609; *p* < 0.001), primary site (OR 1.255; *p* = 0.006), grade (OR 1.255; *p* = 0.009), N stage (OR 1.140; *p* = 0.034), and histology (OR 3.310; *p* = 0.006). In BLM, the significant variables included age (OR 0.523, *p* = 0.002), race (OR 1.394; *p* = 0.020), and radiotherapy (OR 3.648; *p* = 0.043). In PLM, chemotherapy (*p* < 0.001) was an independent factor associated with a survival benefit from PTR.

**Table 2:** Univariate analysis and binary logistic regression of primary tumor resection benefit factors in patients with liver metastases

	Characteristic	Patients with liver metastases		Univariable analysis		Binary logistic regression	
		Benefit group	Non-benefit group	*X*^2^	*P* value	OR (95% CI)	*P* value
CRLM	**Age**			36.057	< 0.001		< 0.001
	18-49	133(19.9%)	34(10.6%)			Reference	
	50-59	212(31.7%)	67(20.9%)			0.844(0.523-1.363)	0.489
	≥ 60	324(48.4%)	220(68.5%)			0.426(0.278-0.654)	< 0.001
	**Grade**			23.984	< 0.001		0.009
	Grade I	13(1.9%)	10(3.1%)			Reference	
	Grade II	492(73.5%)	190(59.2%)			2.025(0.845-4.854	0.114
	Grade III	66(9.9%)	56(17.4%)			1.043(0.408-2.664)	0.930
	Grade IV	4(0.6%)	6(1.9%)			0.737(0.148-3.679)	0.710
	Unknown	94(14.1%)	59(18.4%)			1.445(0.573-3.644)	0.435
	**N stage**			9.586	0.002		0.034
	N0	222(33.2%)	139(43.3%)			Reference	
	N1-N2	447(66.8%)	182(56%)			1.372(1.024-1.837)	
	**Primary Site**			30.110	< 0.001		0.006
	Right colon	137(20.5%)	106(33.0%)			Reference	
	Left colon	168(25.1%)	95(29.6%)			1.137(0.779-1.661)	0.506
	Rectosigmoid	61(9.1%)	27(8.4%)			1.420(0.829-2.431)	0.202
	Rectum	303(45.3%)	93(29.0%)			1.832(1.272-2.639)	0.001
	**Histology**			21.637	< 0.001		0.006
	Mucinous adenocarcinoma	11(1.6%)	24(7.5%)			Reference	
	Nonmucinous adenocarcinoma	658(98,4%)	297(92.5%)			2.992(1.380-6.488)	
BLM	**Age**			13.584	0.001		0.002
	≥ 60	22(23.4%)	35(41.2%)			Reference	
	18-49	48(51.1%)	21(24.7%)			3.967(1.821-8.640)	0.001
	50-59	24(31.5%)	29(34.1%)			1.791(0.794-4.041)	0.160
	**Race**			8.259	0.016		0.020
	Black	9(9.6%)	20(23.5%)			Reference	
	Others	11(11.7%)	4(4.7%)			7.765(1.760-34.257)	0.007
	White	74(78.7%)	61(71.7%)			2.561(1.029-6.375)	0.043
	**Radiotherapy**			4.135	0.042		0.043
	No/Unknown	83(88.3%)	82(96.5%)			Reference	
	Yes	11(11.7%)	3(3.5%)			4.134(1.048-16.313)	
PLM	**Chemotherapy**			39.033	< 0.001		
	No/Unknown	21(17.4%)	30(68.2%)				
	Yes	100(82.6%)	14(31.8%)				

*CRLM* colorectal cancer liver metastases, *BLM* breast cancer liver metastases, *PLM* pancreatic cancer liver metastases, *OR* odds ratio

### Nomogram Construction and Validation

**Fig. 4:**
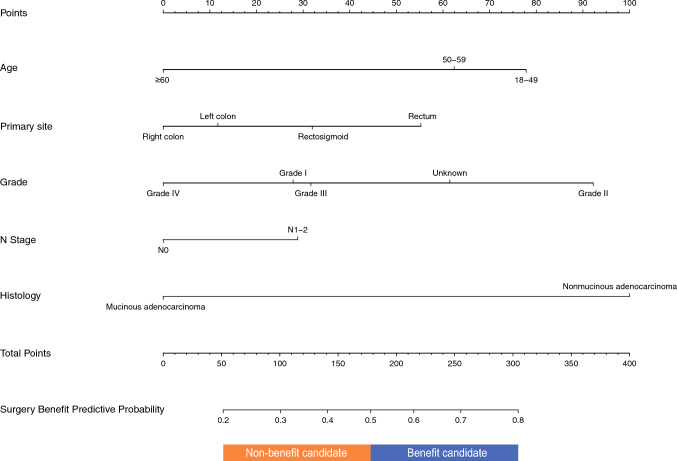
A nomogram was used to identify patients who would benefit from PTR in CRLM. The corresponding scores for each variable were summed to obtain a total score, which was then used to calculate the likelihood of receiving a benefit. Patients with a benefit likelihood of >0.5 were recommended for PTR

To select appropriate candidates for PTR in CRLM, a nomogram was developed for analysis. In CRLM, 1417 patients who underwent PTR were randomly assigned to either a training dataset (70%, *n* = 990) or a validation dataset (30%, *n* = 427). Therefore, in the training cohort, a nomogram of the benefit PTR factors in CRLM patients was established according to the results of binary logical regression, as shown in Fig. 4. To use the nomogram: (1) find the position of each variable on the corresponding axis; (2) draw a vertical line on the point axis to obtain the score of each variable; (3) add the scores of each variable to obtain the score and find it on the total score axis; and (4) draw a vertical line from the total score axis to the ‘surgical benefit prediction probability’ axis to determine the likelihood of liver metastases patients benefiting from PTR. It is recommended that patients with a probability of benefit >0.5 undergo PTR. The C-index of CRLM based on internal and external validation of the training and validation cohorts was 0.678 and 0.699, respectively. The calibration curves indicate the great feasibility of the prediction model, as shown in ESM Fig. 1. ESM Table 5 presents detailed clinical data from 34 CRLM patients who underwent PTR at our center. As shown in ESM Fig. 2, data from this group were used as an external validation set for the nomogram and to construct a calibration curve. In summary, the nomogram can be used to reliably predict benefit from PTR.

## Discussion

This study analyzed a total of 21,928 patients diagnosed with colorectal cancer, pancreatic cancer, breast cancer, and gastric cancer, which are most prone to liver metastasis, to identify the factors that affect the benefit of PTR. In this analysis, the application of PSM reduced the selection bias between the surgery and non-surgery groups. By analyzing the clinicopathological characteristics and using logistic regression, we determined the factors associated with surgical benefits. Additionally, a nomogram was developed specifically for patients with CRLM to help identify suitable candidates for PTR. Internal and external validation ultimately confirmed the reliability of the nomogram in predicting the benefit of PTR.

PTR can reduce tumor-related complications and avoid life-threatening situations. Previous studies have shown that PTR can prolong the survival time of patients with liver metastatic cancer, including colorectal cancer, pancreatic cancer, gastric cancer, and breast cancer.^7–10^ According to Paget’s seed and soil theory,^11^ cancer cells will spread throughout the body circulation when distant metastasis is detected. Therefore, local treatment will not affect the OS rate.^12^ However, there are several other theories that explain the basic principle that PTR increases the OS of stage IV cancer patients. By removing the primary site tumor and reducing the number of tumor cells, the prognosis of patients can be affected.^13^ Removing the primary tumor can reduce the volume of cancer stem cells, thereby improving the efficacy of systemic treatment by reactivating the autoimmune system.^14^ Additionally, the concept of ‘cell vaccination’ indicates that cancer cells released from the primary tumor into the bloodstream will return to the primary tumor and be activated.^15^ These hypothetical mechanisms are all based on basic experimental results; therefore, it is of extreme importance to demonstrate these results clinically.^12^

Multiple studies have shown that the removal of primary lesions is an important factor affecting OS. A study conducted in Canada targeting stage IV colorectal cancer patients found that OS in the surgery group was significantly longer than that in the non-surgery group (27 months vs. 14 months).^16^ In the past decade, the surgical safety of pancreatic cancer has continuously improved, with a mortality rate of <5%, leading to the expansion of local surgical methods for the pancreas. Multiple studies have shown that the median survival time in the surgery group for M1-stage pancreatic ductal adenocarcinoma at the primary site is significantly higher than that in the non-surgery group.^8^ A randomized controlled trial in Turkey showed that PTR, as an initial treatment for newly diagnosed stage IV breast cancer, significantly improved the OS rate. Furthermore, by analyzing the data of 714 patients in three randomized controlled trials, a meta-analysis showed that PTR in stage IV breast cancer also significantly improved the OS rate.^7^ Müsri et al. conducted a retrospective analysis of 288 patients with metastatic gastric cancer and concluded that PTR can prolong the median OS of patients (12.0 months vs. 7.8 months).^10^ These various studies all indicate that PTR can improve the survival of patients with primary tumor metastases, which is partially consistent with our research findings.

For patients with liver metastases, PTR significantly affects survival time and is associated with prognosis. To maximize the effectiveness of surgical treatment, it is important to develop the most appropriate treatment strategy using chemotherapy, surgery, and radiotherapy, alone or in combination.^12^ Due to the potential imbalance in covariate distribution between the surgery and non-surgery groups, there exists a risk of selection bias, with significant heterogeneity observed in clinical data such as age, primary site, and tumor stage. Therefore, 1:1 PSM was employed between the surgery and non-surgery populations to mitigate these mismatches in other clinical information. On this basis, we further investigated the factors that affect surgical benefit. Our research indicates that combining chemotherapy with PTR can result in an improved prognosis for patients with PLM. Chemotherapy after surgical resection is the first-line treatment for PLM patients.^17^ The statistical analysis for GLM patients indicated that performing PTR improved the median CSS, although the difference was not statistically significant. A study by Al-Batran et al.^18^ showed that patients with localized metastatic gastric cancer who received neoadjuvant chemotherapy and underwent surgery showed good survival rates. The median OS time for patients undergoing surgery combined with chemotherapy was 31.3 months, while that of patients receiving chemotherapy only was 15.9 months. Clinical experience shows that PTR for breast cancer can effectively relieve chest symptoms, such as bleeding, ulcers, and pain caused by invasion of the chest wall.^19–24^ Neoadjuvant chemotherapy for operable breast cancer can reduce neoplasm staging as well as axillary lymph node metastasis,^25^ thus improving the cosmetic effect and reducing the incidence rate of surgery. Some studies have shown that factors such as ‘complete resection of primary tumor’, ‘new metastasis’, and being ‘young’ indicate that patients are suitable candidates for breast cancer PTR,^21,22^ which is consistent with some of our research results. Our study further confirms that factors associated with PTR benefits in BLM include younger age, race, and receiving radiotherapy.

In patients with CRLM, there are several factors that affect the benefits of PTR. Personalized clinical decisions should be made with comprehensive consideration. From an embryological perspective, the view that the OS of left colorectal cancer patients is greater than that of right colorectal cancer patients has been widely proven and explained. They have different origins, therefore they have differences in incidence,^26,27^ in clinical, endoscopic and histological appearances,^28,29^ and in some molecular characteristics.^30,31^ It is obvious that patients with liver metastases from right colon cancer have the worst survival rate and the least chance of benefiting from surgery in our study. The results show that patients with younger age, lower pathological grade, and N1 stage are more eligible for surgery. Based on these significant variables, we developed a nomogram for CRLM patients that significantly enhances clinical utility. Furthermore, the nomogram also performed well in both internal and external validation.

This study has some limitations. This was a retrospective study based on the SEER database, which introduces inherent biases, such as the absence of detailed information on treatment and complications, number of liver metastases, and molecular profiling. PSM has minimized these differences between groups but it lacks specific details regarding the type and duration of chemotherapy and radiotherapy. Therefore, we can only classify patients as receiving chemotherapy, radiotherapy, or ‘none/unknown’, potentially introducing bias.

## Conclusion

PTR significantly influenced the survival outcomes in CRLM, BLM, and PLM patients. We conducted an analysis of factors influencing the benefit of PTR and developed a predictive model based on the CRLM population. The nomogram was validated to quantify the benefits of PTR, aiding in the selection of optimal PTR candidates among patients with CRLM, without additional costs and with potential prognostic benefits.

## Supplementary Information

Below is the link to the electronic supplementary material.

Supplementary file1 (DOCX 18 KB)

Supplementary file2 (DOCX 18 KB)

Supplementary file3 (DOCX 20 KB)

Supplementary file4 (DOCX 20 KB)

Supplementary file5 (DOCX 16 KB)

Supplementary file6 (EPS 3425 KB)

Supplementary file7 (EPS 1045 KB)

## Data Availability

Data files from the SEER database were downloaded directly from the SEER website (https://seer.cancer.gov/.) The relevant data from Shanghai East Hospital are available from the corresponding author upon reasonable request.
