# Supplementary material for: The Value of Primary Tumor Resection in Patients with Liver Metastases: A 10-Year Outcome
Source: Ann Surg Oncol. 2024 Nov 4;32(2):1083–92. doi: 10.1245/s10434-024-16386-3 (PMC11698763; doi:10.1245/s10434-024-16386-3)
Supplement: Supplementary file 1 — Supplementary file1 (DOCX 18 KB) [file 10434_2024_16386_MOESM1_ESM.docx]

**Supplement Table 1** Demographic information for patients with colorectal cancer with liver metastases before and after propensity score matching

| Characteristic | Before PSM | |  | After PSM | | |
| --- | --- | --- | --- | --- | --- | --- |
|  | Patients  with surgery | Patients  without surgery | *P* value | Patients  with surgery | Patients  without surgery | *P* value |
|  | (n=10505), n (%) | (n=4436), n (%) |  | (n=1417), n (%) | (n=1417), n (%) |  |
| **Age** |  |  | 0.031 |  |  | 0.999 |
| 18-49 | 1863(17.7) | 733(16.5) |  | 241(17.0) | 240(16.9) |  |
| 50-59 | 2692(25.6) | 1218(27.5) |  | 389(27.5) | 389(27.5) |  |
| ≥60 | 5950(56.7) | 2485(56.0) |  | 787(55.5) | 788(55.6) |  |
| **Histology** |  |  | 0.349 |  |  | 0.575 |
| Nonmucinous adenocarcinoma | 9395(89.4) | 3990(89.9) |  | 1363(96.2) | 1355(95.6) |  |
| Mucinous adenocarcinoma | 1110(10.6) | 446(10.1) |  | 54(3.8) | 62(4.4) |  |
| **Race** |  |  | 0.628 |  |  | 0.993 |
| White | 7907(75.3) | 3306(74.5) |  | 1164(82.1) | 1162(82.0) |  |
| Black | 1618(15.4) | 706(15.9) |  | 161(11.4) | 163(11.5) |  |
| Others | 980(9.3) | 424(9.6) |  | 92(6.5) | 92(6.5) |  |
| **Gender** |  |  | <0.001 |  |  | 0.785 |
| Female | 4683(44.6) | 1809(40.8) |  | 522(36.8) | 529(37.3) |  |
| Male | 5822(55.4) | 2627(59.2) |  | 895(63.2) | 888(62.7) |  |
| **Grade** |  |  | <0.001 |  |  | 0.995 |
| Grade I | 403(3.8) | 191(4.3) |  | 39(2.8) | 40(2.8) |  |
| Grade II | 6836(65.1) | 2054(46.3) |  | 962(67.9) | 955(67.4) |  |
| Grade III | 2283(21.7) | 651(14.7) |  | 188(13.3) | 195(13.8) |  |
| Grade IV | 549(5.3) | 76(1.7) |  | 14(0.9) | 13(0.9) |  |
| Unknown | 434(4.1) | 1464(33.0) |  | 214(15.1) | 214(15.1) |  |
| **T stage** |  |  | <0.001 |  |  | 0.973 |
| T0 | 1(0.0) | 24(0.5) |  |  |  |  |
| T1 | 237(2.3) | 1870(42.2) |  | 163(11.5) | 163(11.5) |  |
| T2 | 322(3.0) | 125(2.8) |  | 23(1.6) | 20(1.4) |  |
| T3 | 6036(57.5) | 1316(29.7) |  | 784(55.3) | 783(55.3) |  |
| T4 | 3909(37.2) | 1101(24.8) |  | 447(31.6) | 451(31.8) |  |
| **N stage** |  |  | <0.001 |  |  | 1.000 |
| N0 | 1800(17.2) | 2324(52.4) |  | 524(36.9) | 524(36.9) |  |
| N1 | 3912(37.2) | 1791(40.4) |  | 732(51.7) | 732(51.7) |  |
| N2 | 4793(45.6) | 321(7.2) |  | 161(11.4) | 161(11.4) |  |
| **Primary Site** |  |  | <0.001 |  |  | 1.000 |
| Right colon | 4726(44.9) | 1219(27.5) |  | 362(25.5) | 363(25.6) |  |
| Left colon | 3641(34.7) | 1024(23.1) |  | 365(25.8) | 363(25.6) |  |
| Rectosigmoid | 965(9.2) | 475(10.7) |  | 138(9.7) | 137(9.7) |  |
| Rectum | 1173(11.2) | 1718(38.7) |  | 552(39.0) | 554(39.1) |  |
| **Chemotherapy** |  |  | 0.218 |  |  | 0.925 |
| No/Unknown | 3016(28.7) | 1318(29.7) |  | 283(20.0) | 285(20.1) |  |
| Yes | 7489(71.3) | 3118(70.3) |  | 1134(80.0) | 1131(79.9) |  |
| **Radiotherapy** |  |  | <0.001 |  |  | 0.673 |
| No/Unknown | 9653(91.9) | 3624(81.7) |  | 1138(80.3) | 1129(79.7) |  |
| Yes | 852(8.1) | 812(18.3) |  | 279(19.7) | 288(20.3) |  |
| PSM, propensity score matching. | | | | | | |
