# Supplementary material for: The Value of Primary Tumor Resection in Patients with Liver Metastases: A 10-Year Outcome
Source: Ann Surg Oncol. 2024 Nov 4;32(2):1083–92. doi: 10.1245/s10434-024-16386-3 (PMC11698763; doi:10.1245/s10434-024-16386-3)
Supplement: Supplementary file 2 — Supplementary file2 (DOCX 18 KB) [file 10434_2024_16386_MOESM2_ESM.docx]

**Supplement Table 2** Demographic information for patients with breast cancer with liver metastases before and after propensity score matching

| Characteristic | Before PSM |  |  | After PSM |  |  | |
| --- | --- | --- | --- | --- | --- | --- | --- |
|  | Patients with surgery | Patients without surgery | P value | Patients with surgery | Patients without surgery | P value | |
|  | (n=302), n (%) | (n=360), n (%) |  | (n=179), n (%) | (n=179), n (%) |  | |
| **Age** |  |  | 0.014 |  |  | 0.904 | |
| 18-49 | 118 (39.1) | 106 (29.4) |  | 69 (38.5) | 66 (36.9) |  | |
| 50-59 | 87 (28.8) | 104 (28.9) |  | 53 (29.6) | 55 (30.7) |  | |
| ≥60 | 97 (32.1) | 150 (41.7) |  | 57 (31.9) | 58 (32.4) |  | |
| **Race** |  |  | 0.929 |  |  | 0.731 | |
| Black | 51 (16.9) | 64 (17.8) |  | 29 (16.2) | 28 (15.6) |  | |
| White | 224 (74.2) | 266 (73.9) |  | 135 (75.4) | 135 (75.5) |  | |
| Others | 27 (8.9) | 30 (8.3) |  | 15 (8.4) | 16 (8.9) |  | |
| **Histology** |  |  | 0.009 |  |  | 0.158 | |
| Infiltrating duct carcinoma | 293 (97.0) | 331 (91.9) |  | 174 (97.2) | 172 (96.1) |  | |
| Lobular carcinoma | 9 (3.0) | 29 (8.1) |  | 5 (2.8) | 7 (3.9) |  | |
| **T Stage** |  |  | 0.248 |  |  | 0.875 | |
| T0-1 | 49 (16.2) | 59 (16.4) |  | 35 (19.6) | 27 (15.1) |  | |
| T2 | 122 (40.4) | 139 (38.6) |  | 70 (39.1) | 72 (40.2) |  | |
| T3 | 71 (23.5) | 69 (19.2) |  | 40 (22.3) | 41 (22.9) |  | |
| T4 | 60 (19.9) | 93 (25.8) |  | 34 (19.0) | 39 (21.8) |  | |
| **N Stage** |  |  | <0.001 |  |  | 0.972 | |
| N0 | 51 (16.9) | 89 (24.7) |  | 41 (22.9) | 35 (19.6) |  | |
| N1 | 134 (44.4) | 204 (56.7) |  | 87 (48.6) | 92 (51.4) |  | |
| N2 | 60 (19.9) | 28 (7.8) |  | 25 (14.0) | 22 (12.2) |  | |
| N3 | 57 (18.8) | 39 (10.8) |  | 26 (14.5) | 30 (16.8) |  | |
| **Radiotherapy** |  |  | <0.001 |  |  | 0.949 | |
| No/Unknown | 190 (62.9) | 342 (95.0) |  | 165 (92.2) | 162 (90.5) |  | |
| Yes | 112 (37.1) | 18 (5.0) |  | 14 (7.8) | 17 (9.5) |  | |
| **Chemotherapy** |  |  | <0.001 |  |  | 1.000 | |
| No/Unknown | 50 (16.6) | 119 (33.1) |  | 39 (21.8) | 46 (25.7) |  | |
| Yes | 252 (83.4) | 241 (66.9) |  | 140 (78.2) | 133 (74.3) |  | |
| PSM, propensity score matching. | | | | | | |  |
