# Supplementary material for: The Value of Primary Tumor Resection in Patients with Liver Metastases: A 10-Year Outcome
Source: Ann Surg Oncol. 2024 Nov 4;32(2):1083–92. doi: 10.1245/s10434-024-16386-3 (PMC11698763; doi:10.1245/s10434-024-16386-3)
Supplement: Supplementary file 3 — Supplementary file3 (DOCX 20 KB) [file 10434_2024_16386_MOESM3_ESM.docx]

**Supplement Table 3** Demographic information for patients with gastric cancer with liver metastases before and after propensity score matching

| Characteristic | Before PSM |  |  | After PSM |  |  | |
| --- | --- | --- | --- | --- | --- | --- | --- |
|  | Patients with surgery | Patients without surgery | *P* value | Patients with surgery | Patients without surgery | *P* value | |
|  | (n=129), n (%) | (n=953), n (%) |  | (n=84), n (%) | (n=84), n (%) |  | |
| **Age** |  |  | 0.119 |  |  | 1.000 | |
| 18-65 | 50 (38.8) | 443 (46.5) |  | 37 (44.0) | 36 (42.9) |  | |
| ≥65 | 79 (61.2) | 668 (53.5) |  | 47 (56.0) | 48(45.1) |  | |
| **Race** |  |  | 0.015 |  |  | 0.342 | |
| Black | 23 (17.8) | 138 (14.5) |  | 16 (19.0) | 13 (15.5) |  | |
| White | 79 (61.3) | 693 (72.7) |  | 57 (67.9) | 53 (63.1) |  | |
| Other | 27 (20.9) | 122 (12.8) |  | 11 (13.1) | 18 (21.4) |  | |
| **Gender** |  |  | 0.819 |  |  | 0.393 | |
| Female | 37 (28.7) | 260 (27.3) |  | 27 (32.1) | 21 (25.0) |  | |
| Male | 92 (71.3) | 693 (72.7) |  | 57 (67.9) | 63 (75.0) |  | |
| **Primary Site** |  |  | <0.001 |  |  | 0.879 | |
| Body | 12 (9.3) | 79 (8.3) |  | 10 (11.9) | 5 (6.0) |  | |
| Cardia | 25 (19.4) | 414 (43.4) |  | 22 (26.2) | 24 (28.6) |  | |
| Fundus | 3 (2.3) | 36 (3.8) |  | 3 (3.6) | 2 (2.4) |  | |
| Gastric antrum | 42 (32.6) | 122 (12.8) |  | 20 (23.8) | 22 (26.2) |  | |
| Greater | 4 (3.1) | 38 (4.0) |  | 4 (4.8) | 4 (4.8) |  | |
| Lesser | 12 (9.3) | 57 (6.0) |  | 6 (7.1) | 4 (4.8) |  | |
| Others | 25 (19.4) | 188 (19.7) |  | 16 (19.0) | 18 (21.2) |  | |
| Pylorus | 6 (4.6) | 19 (2.0) |  | 3 (3.6) | 5 (6.0) |  | |
| **Histology** |  |  | 0.453 |  |  | 1.000 | |
| Adenocarcinoma | 114 (88.4) | 866 (90.9) |  | 75 (89.3) | 74 (88.1) |  | |
| Signet ring cell carcinoma | 15 (11.6) | 87 (9.1) |  | 9 (10.7) | 10 (11.9) |  | |
| **T Stage** |  |  | <0.001 |  |  | 0.914 | |
| T0-T1 | 9 (7.0) | 436 (45.8) |  | 9 (10.7) | 7 (8.3) |  | |
| T2 | 6 (4.7) | 45 (4.7) |  | 5 (6.0) | 6 (7.1) |  | |
| T3 | 60 (46.5) | 211 (22.1) |  | 36 (42.9) | 39 (46.4) |  | |
| T4 | 54 (41.8) | 261 (27.4) |  | 34 (40.4) | 32 (38.2) |  | |
| **N Stage** |  |  | <0.001 |  |  | 0.949 | |
| N0 | 27 (20.9) | 411 (43.1) |  | 25 (29.8) | 25 (29.8) |  | |
| N1 | 30 (23.3) | 451 (47.3) |  | 28 (33.3) | 30 (35.7) |  | |
| N2 | 31 (24.0) | 58 (6.1) |  | 20 (23.8) | 17 (20.2) |  | |
| N3 | 41 (31.8) | 33 (3.5) |  | 11 (13.1) | 12 (14.3) |  | |
| **Radiotherapy** |  |  | 0.234 |  |  | 1.000 | |
| No/Unknown | 112 (86.8) | 783 (82.2) |  | 69 (82.1) | 68 (81.0) |  | |
| Yes | 17 (13.2) | 170 (17.8) |  | 15 (17.9) | 16 (19.0) |  | |
| **Chemotherapy** |  |  | <0.001 |  |  | 0.439 | |
| No/Unknown | 72 (55.8) | 372 (39.0) |  | 42 (50.0) | 36 (42.9) |  | |
| Yes | 57 (44.2) | 581 (61.0) |  | 42 (50.0) | 48 (57.1) |  | |
| PSM, propensity score matching. | | | | | | |  |
