# Supplementary material for: The Value of Primary Tumor Resection in Patients with Liver Metastases: A 10-Year Outcome
Source: Ann Surg Oncol. 2024 Nov 4;32(2):1083–92. doi: 10.1245/s10434-024-16386-3 (PMC11698763; doi:10.1245/s10434-024-16386-3)
Supplement: Supplementary file 4 — Supplementary file4 (DOCX 20 KB) [file 10434_2024_16386_MOESM4_ESM.docx]

**Supplement Table 4** Demographic information for patients with pancreatic cancer with liver metastases before and after propensity score matching

| Characteristic | Before PSM |  |  | After PSM |  |  |  | |
| --- | --- | --- | --- | --- | --- | --- | --- | --- |
|  | Patients with surgery | Patients without surgery | *P* value | Patients with surgery | Patients without surgery | *P* value |  | |
|  | (n=174), n (%) | (n=5,069), n(%) |  | (n=165), n (%) | (n=165), n (%) |  |  | |
| **Age** |  |  | 0.004 |  |  | 0.564 |  | |
| 18-49 | 22 (12.6) | 327 (6.5) |  | 18 (10.9) | 14 (8.5) |  |  | |
| 50-59 | 40 (23.0) | 1134 (22.4) |  | 37 (22.4) | 44 (26.7) |  |  | |
| ≥60 | 112 (64.4) | 3608 (71.1) |  | 110 (66.7) | 107 (64.8) |  |  | |
| **Race** |  |  | 0.412 |  |  | 0.962 |  | |
| White | 138 (79.3) | 4004 (79.0) |  | 131 (79.4) | 133 (80.6) |  |  | |
| Black | 20 (11.5) | 706 (13.9) |  | 19 (11.5) | 18 (10.9) |  |  | |
| Others | 16 (9.2) | 359 (7.1) |  | 15 (9.1) | 14 (8.5) |  |  | |
| **Gender** |  |  | 0.301 |  |  | 1.000 |  | |
| Female | 85 (48.9) | 2260 (44.6) |  | 81 (49.1) | 82 (49.7) |  |  | |
| Male | 89 (51.1) | 2809 (55.4) |  | 84 (50.9) | 83 (50.3) |  |  | |
| **Primary site** |  |  | <0.001 |  |  | 0.706 |  | |
| Body | 15 (8.6) | 848 (16.7) |  | 15 (9.1) | 14 (8.5) |  |  | |
| Head | 105 (60.3) | 2007 (39.6) |  | 101 (61.2) | 105 (63.6) |  |  | |
| Tail | 41 (23.6) | 1149 (22.7) |  | 36 (21.8) | 29 (17.6) |  |  | |
| Others | 13 (7.5) | 1065 (21.0) |  | 13 (7.9) | 17 (10.3) |  |  | |
| **Grade** |  |  | <0.001 |  |  | 0.777 |  | |
| Grade I-Grade II | 69 (39.7) | 547 (10.8) |  | 62 (37.6) | 66 (40.0) |  |  | |
| Grade III-Grade IV | 69 (39.7) | 649 (12.8) |  | 67 (40.6) | 68 (41.2) |  |  | |
| Unknown | 36 (20.6) | 3873 (76.4) |  | 36 (21.8) | 31 (18.8) |  |  | |
| **T Stage** |  |  | <0.001 |  |  | 0.693 |  | |
| T0-T1 | 7 (4.0) | 237 (4.7) |  | 7 (4.2) | 4 (2.4) |  |  | |
| T2 | 26 (14.9) | 2036 (40.2) |  | 26 (15.8) | 30 (18.2) |  |  | |
| T3 | 126 (72.4) | 1705 (33.6) |  | 117 (70.9) | 119 (72.1) |  |  | |
| T4 | 15 (8.7) | 1091 (21.5) |  | 15 (9.1) | 12 (7.3) |  |  | |
| **N Stage** |  |  | <0.001 |  |  | 1.000 |  | |
| N0 | 63 (36.2) | 3285 (64.8) |  | 63 (38.2) | 64 (38.8) |  |  | |
| N1 | 111 (63.8) | 1784 (35.2) |  | 102 (61.8) | 101 (61.2) |  |  | |
| **Radiotherapy** |  |  | 0.005 |  |  | 0.345 |  | |
| No/Unknown | 161 (92.5) | 4904 (96.7) |  | 153 (92.7%) | 158 (95.8) |  |  | |
| Yes | 13 (7.5) | 165 (3.3) |  | 12 (7.3%) | 7 (4.2) |  |  | |
| **Chemotherapy** |  |  | 0.005 |  |  | 0.813 |  | |
| No/Unknown | 52 (29.9) | 2073 (40.9) |  | 51 (30.9) | 54 (32.7) |  |  | |
| Yes | 122 (70.1) | 2996 (59.1) |  | 114 (69.1) | 111 (67.3) |  |  | |
| PSM, propensity score matching. | | | | | | | |  |
