# Supplementary material for: The Value of Primary Tumor Resection in Patients with Liver Metastases: A 10-Year Outcome
Source: Ann Surg Oncol. 2024 Nov 4;32(2):1083–92. doi: 10.1245/s10434-024-16386-3 (PMC11698763; doi:10.1245/s10434-024-16386-3)
Supplement: Supplementary file 5 — Supplementary file5 (DOCX 16 KB) [file 10434_2024_16386_MOESM5_ESM.docx]

**Supplement Table 5** Demographic information for patients with colorectal cancer with liver metastases in our center

| **Characteristic** | **(n=34)** |
| --- | --- |
| **Age** |  |
| 18-49  50-59  ≥60 | 10 (29.4%)  5 (14.7%)  19 (55.9%) |
| **Gender** |  |
| Female | 14 (41.2%) |
| Male | 20 (58.8%) |
| **Primary Site** |  |
| Right colon | 7 (20.6%) |
| Left colon  Rectosigmoid  Rectum  **Histology**  Nonmucinous adenocarcinoma  **Grade**  Grade II  Grade III  Grade IV  Unknown | 8 (23.5%)  3 (8.8%)  16 (47.1%)  34 (100.0%)  25(73.5%)  4(11.8%)  1(2.9%)  4 (11.8%) |
| **T Stage** |  |
| T1-T2 | 5 (14.7%) |
| T3-T4 | 29 (85.3%) |
| **N Stage** |  |
| N0 | 8 (23.5%) |
| N1-N2 | 26 (76.5%) |
| **Radiotherapy** |  |
| No/Unknown  Yes | 26 (76.5%)  8 (23.5%) |
| **Chemotherapy**  No/Unknown | 4 (11.8%) |
| Yes | 30 (88.2%) |
